# A randomised, double-blind, active placebo-controlled, parallel groups, dose-response study of scopolamine hydrobromide (4–6 μg/kg) in patients with major depressive disorder

**DOI:** 10.1186/s13063-020-4089-6

**Published:** 2020-02-10

**Authors:** Joseph C. C. Chen, Rachael L. Sumner, Venkat Krishnamurthy Naga, Nicholas Hoeh, Hafis Adetokunbo Ayeni, Vikrant Singh, Frederick Sundram, Douglas Campbell, Suresh Muthukumaraswamy

**Affiliations:** 1 School of Pharmacy, Faculty of Medical and Health Sciences, University of Auckland, 85 Park Road, Grafton, Auckland, 1023 New Zealand; 2 Acute Adult North Community Mental Health Services, Waitemata District Health Board, 44 Taharoto Road, Takapuna, Auckland, 0622 New Zealand; 3 Department of Psychological Medicine, School of Medicine, Faculty of Medical and Health Sciences, University of Auckland, 2 Park Road, Grafton, Auckland, 1023 New Zealand; 4 Department of Anaesthesiology and Perioperative Medicine, Auckland City Hospital, 2 Park Road, Grafton, Auckland, 1023 New Zealand

**Keywords:** Scopolamine, Depression, EEG, Pharmacokinetic, Pharmacodynamic

## Abstract

**Background:**

Depressive disorders are a leading cause of disability, but current behavioural and pharmacological therapies have a slow onset of response, typically taking several weeks before achieving efficacy. Prior studies using triplicate intravenous scopolamine infusions have been shown to reduce depressive symptomologies within days compared to saline placebo infusions. However, several parameters of scopolamine’s potential antidepressant effect remain unknown, such as its dose–response profile and its washout period. There is also the question as to whether the previously reported antidepressant responses were confounded by unblinding effects due to the lack of an active placebo control. Glycopyrronium bromide was selected as placebo for this trial given it has similar antimuscarinic properties to scopolamine hydrobromide but an inability to cross the blood–brain barrier, thereby hypothetically mimicking only the peripheral effects of scopolamine.

**Methods/Design:**

A parallel group trial of single intravenous scopolamine infusions at three doses (4, 5, and 6 μg/kg) along with one glycopyrronium bromide 4 μg/kg group will be administered to 40 participants with major depressive disorder in a 1:1:1:2 ratio, respectively. The primary outcome measure will be the Montgomery–Åsberg Depression Rating Scale (MADRS) administered at baseline, 4 hours, 1 day, 3 days, 1 week, 2 weeks, 4 weeks, and 6 weeks post-infusion to determine antidepressant efficacy. As a secondary measure, the Quick Inventory of Depressive Symptomatology will be administered alongside the MADRS to further track potential antidepressant responses. Other secondary measures include electroencephalography, blood samples, and Bowdle visual acuity scales recorded at baseline, 5, 10, 15, 20, 30, 60, 120, and 240 min post-infusion to determine the pharmacokinetic-pharmacodynamic profile of scopolamine in depressed participants.

**Discussion:**

This trial contributes to the literature surrounding the efficacy of scopolamine as an antidepressant. Determining the dose–response profile and washout period of scopolamine’s antidepressant effect will also provide important information for designing and conducting crossover trials. The use of an active placebo is important to reduce potentially confounding expectancy effects.

**Trial registration:**

The trial was registered in the Australian New Zealand Clinical Trials Registry (registration number ACTRN12619000569101). Registered on 11 April 2019.

## Background

Depression is the leading cause of disability globally with over 300 million individuals affected worldwide [1]. Defined in the *Diagnostic and Statistical Manual of Mental Disorders* (DSM-V) as depressed mood and/or loss of interest or pleasure, depression has diverse and debilitating effects on daily functioning [2]. In severe cases, depression can be life-threatening and death due to suicide can occur, with approximately 800,000 suicide-deaths recorded each year [1]. Despite the large range of available behavioural and pharmacological therapies, approximately one-third of patients do not achieve remission even after trying four or more antidepressant medications [3]. Furthermore, present first-line pharmacotherapies have a slow onset of response (typically 4–6 weeks) and may have undesirable side effects (such as increasing suicidality in paediatric populations) [4]—both of which are particularly problematic with depressed patients experiencing suicidal ideation. A clear need therefore exists for the development of novel and rapid acting therapies.

An initial study established the antidepressant properties of intravenously delivered scopolamine at doses of 2–4 μg/kg in volunteers with major depressive disorder (MDD) and bipolar depression [5]. The same research group replicated the findings in an independent patient sample consisting only of unipolar depressed patients [6]. By pooling data from the previous two studies and recruiting additional participants, the authors found slightly larger antidepressant effects in women [7] along with antidepressant effects in both treatment-naïve and treatment-resistant depressed patients [8]. However, a recent study utilising an independent participant sample found no significant antidepressant effect compared to placebo—though the authors noted that their population was more severely depressed and treatment-resistant than previous studies [9]. To determine scopolamine’s antidepressant efficacy, these prior studies all utilised the same experimental design: a double-blind, saline placebo-controlled, crossover clinical trial with a single-blind lead-in session. The infusion regimen dosed participants in two blocks of either triplicate infusions of 4 μg/kg scopolamine hydrobromide or triplicate infusions of saline placebo with each infusion being temporally separated by 3–4 days. Participants were clinically assessed via the Montgomery–Åsberg Depression Rating Scale (MADRS) on each infusion day along with a follow-up session 3–4 days after the last infusion day.

However, there remain questions regarding scopolamine’s antidepressant efficacy, the optimal scopolamine dose, and the duration of scopolamine’s antidepressant effect. A particular need exists for replication and evaluation of scopolamine’s efficacy by an independent research group, especially given the recent failure to replicate the antidepressant response in an independent patient sample [9]. Furthermore, the earliest paper utilised intravenous scopolamine doses ranging from 2 to 4 μg/kg where the 4 μg/kg dose was deemed most efficacious in eliciting an antidepressant response [5]. Subsequent studies continued to use the 4 μg/kg dosing regimen and, as such, it is currently unknown whether higher doses may be more efficacious.

To date, a crossover design has been used which was too short (3–4 days) to adequately account for carryover effects [5–9]. The antidepressant effects of scopolamine from the first block of triplicate infusions are clearly evident in the psychiatric measures of antidepressant response leading into the second block of triplicate saline placebo infusions [5–9]. Not only does this introduce an unwanted carryover effect in the placebo data, but it also does not characterise the expected antidepressant duration of scopolamine, a parameter established in other rapid acting antidepressants such as ketamine [10]. Determining the duration of scopolamine’s antidepressant response is critical to designing future crossover trials. Further to this, the use of a triplicate infusion protocol across 9–12 days has never been established as necessary. A single infusion at 4 μg/kg or higher may be just as effective. The requirement for triplicate infusions places considerable demand on the participants in clinical trials as well as future demand on clinicians and patients should scopolamine become an approved therapy. Should the present protocol be successful in determining an antidepressant effect, there will be a reduction in participant time commitments.

A further key consideration of future research into scopolamine’s antidepressant efficacy is maintaining blinding. Alongside measuring the antidepressant effects of scopolamine, other psychotropic, neurological, and physiological effects have also been observed in healthy controls. For example, significant changes to feeling high, alertness, feelings of ‘internal perception’ and ‘external perception’ compared to placebo have been found in healthy male individuals [11]. Given the significant psychotropic effects characterised in healthy individuals compared to saline placebo, there may be concerns regarding unblinding effects in participants. This is particularly important in an antidepressant trial where the present primary outcome is a mood-related measure, as issues with substantial placebo responses in antidepressant trials are well documented [12].

Past studies have characterised pharmacokinetic and pharmacodynamic properties of intravenous, intramuscular, and subcutaneous scopolamine, demonstrating that electroencephalography (EEG) alpha power significantly decreases acutely after administration compared to saline placebo [13, 14]. EEG measures may provide insight into the antidepressant mechanisms of scopolamine, as resting-state spectral features particularly in the alpha band (8–12 Hz) have been related to the antidepressant properties of other antidepressants such as selective serotonin reuptake inhibitors (SSRIs) [15, 16]. A number of studies have shown that ketamine [17–20] and scopolamine [13] both cause rapid decreases in the power of the EEG alpha rhythm, which may potentially relate to both resting-state network connectivity decreases and also the antidepressant response to these drugs.

The current study aims to extend the existing literature as the first independent replication of scopolamine hydrobromide as an antidepressant following several foundational studies [5–9]. Furthermore, this study will investigate whether doses above 4 μg/kg are more effective, and whether single doses are sufficient to elicit an antidepressant response. As a further aim, the duration of antidepressant response to scopolamine will be determined in order to inform the design of future crossover trials that better control for carryover effects. Blinding in randomised controlled trials of antidepressant therapies is of critical importance for overcoming the well-known issue of substantial placebo response in research into antidepressant efficacy. As such, the present study will use glycopyrrolate as the active placebo as it is a muscarinic antagonist that does not cross the blood–brain barrier and should consequently mimic the peripheral muscarinic effects of scopolamine leading to superior blinding to saline (as previously used) [5–9].

## Methods/Design

### Participants

Volunteers will be adults diagnosed with major depressive disorder, in otherwise good health, and not on antidepressants. The full inclusion and exclusion criteria are outlined in Tables 1 and 2.

**Table 1:** Full inclusion criteria

	Inclusion criteria
Consent	Willing and able to give informed consent for participation in the trial
Demographics	
Age	18–60 years
Sex	Male or female
Mental health	
Diagnosis	Major depressive disorder according to DSM-V criteria
Duration	Greater than 2 weeks
MADRS	≥ 20 (i.e., moderate to severe depression)
Treatment status	Antidepressant medication free for at least 2 weeks (or 4 weeks if previously on fluoxetine)

**Table 2:** Full exclusion criteria

	Exclusion criteria
Consent/communication	Inability to speak or read English
Mental health	
Lifetime	History of psychotic disorder
Current	Any unstable medical or neurologic condition, judged at the discretion of the clinician
	Imminent risk of suicide as determined by the MADRS/clinical interview
	Substance abuse or dependence in previous 3 months
	Stage 3 treatment-resistant depression or higher as determined by Thase and Rush Staging criteria [21]
	Receiving neuromodulation treatment
	Undergoing planned changes to psychotropic medication
Drug contraindications	Significant renal or hepatic impairment
	Cardiovascular conditions including abnormal heart rate and blood pressure checked at screening
	Glaucoma
	Female participants who are pregnant, lactating, or planning pregnancy during the course of the trial
	Contraindication to the use of scopolamine or glycopyrrolate according to manufacturer guidelines
	Regular use of any medication deemed to be contraindicated as judged by the attending study physicians
Other safety criteria	Inability to fast for 2 hours prior to each administration of trial drug
	Any other condition judged by the study clinicians as likely to impact on the ability of the participant to complete the trial
	Are currently attending a New Zealand specialist mental health or addiction service

### Study design

This is a randomised, double-blinded, active placebo-controlled parallel groups trial primarily occurring at the Clinical Research Centre of the Faculty of Medical and Health Sciences, University of Auckland. At an initial screening visit, patients with MDD will give informed consent and be checked for physical and psychiatric eligibility. Participants will be randomly allocated to one of four groups (4 μg/kg glycopyrronium bromide or 4 μg/kg or 5 μg/kg or 6 μg/kg scopolamine hydrobromide) in a 2:1:1:1 allocation ratio with a total desired sample size of 40 (see “Statistical analyses and power calculations” section below). The drug will be administered by an intravenous line controlled by an infusion pump (Alaris PK, UK), programmed by a supervising medical doctor over 15 min. The primary psychiatric outcome will be measured by the clinician-administered MADRS [22] at several time-points: pre-infusion baseline, post-infusion 4 hours, 1 day, 3 days, 1 week, 2 weeks, 4 weeks, and 6 weeks. For time-points from day 1 and onwards, the MADRS will be administered via telephone interview.

Secondary outcome measures will include the self-report Quick Inventory of Depressive Symptomatology (QIDS) [23], which will be co-administered at the same time points of every MADRS. Further secondary physiological and psychotropic outcomes will be measured through resting-state eyes-open EEG scans, 10 mL blood samples, and the Bowdle visual acuity scales (VAS) [24] administered pre-infusion and post-infusion at 5, 10, 15, 20, 30, 60, 120, and 240 min. Further resting-state EEG scans will be performed at 75, 90, 105, 150, 180, and 205 min post-infusion. To monitor the drug’s adverse effect profile, the Generic Assessment of Side Effects (GASE) questionnaire [25] will also be completed at 3 h and 1 week post-infusion. The nine blood samples will be acquired through a cannula in the antecubital fossa into Vacutainer® tubes containing ethylenediaminetetraacetic acid (EDTA; (Benton Dickinson, New Jersey, USA) and stored at 4 °C for 1–6 h. The blood samples will then be centrifuged at 2000 g at 4 °C for 15 min and plasma will be retrieved in 500 μL aliquots. The buffy coat layer will be collected for DNA extraction in order to analyse whether genes related to brain and liver function modify the response to applied interventions. The pharmacokinetic scopolamine concentrations in plasma will be determined using an assay based off a previously validated protocol [26]. The pharmacodynamic EEG scans and Bowdle VAS will be used to characterise the acute effects of a scopolamine infusion.

For exploratory analyses, other questionnaires used during the study day will include the Subjective High Assessment Scale (SHAS) [27], Biphasic Alcohol Effect Scale (BAES) [28], Clinician-Administered Dissociative States Scale (CADSS) [29], and the 5-Dimensional Altered State of Consciousness (5D-ASC) [30] to assess potential sedative as well as psychoactive effects of scopolamine. To add to this, a semi-structured qualitative interview will extensively characterise each individual participant’s psychotropic response to scopolamine. The Credibility Expectancy Questionnaire (CEQ) [31] will be used to test treatment expectancy effects. Participants will also be given a “Charge 3” fitness tracker (Fitbit, San Francisco, CA, USA) to wear for at least a week prior to and until 6 weeks after the drug infusion study day in order to track changes in sleep and activity. This is due to prior investigations showing changes to rapid eye movement sleep characteristics such as onset time and latency along with modest antidepressant effects via the profile of mood states depression subscale [32]. Given scopolamine’s sedative effects and the correlational effects between depressive mood and poor sleep [33], it is also possible that scopolamine’s antidepressant effects are manifested through improved or corrected sleep mechanisms. It is therefore of interest to track participant sleep quality throughout the present trial. Changes in activity (such as increased steps) will be used to corroborate self-report of energy and lethargy in the QIDS and MADRS.

**Fig. 1:**
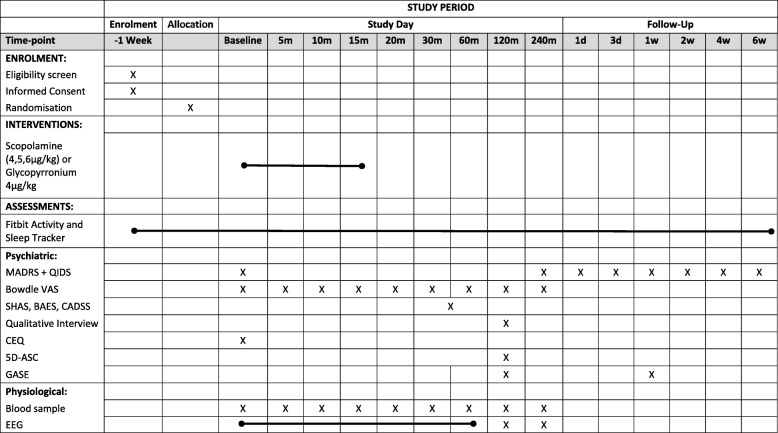
Standard Protocol Items Recommendations for Interventional Trials (SPIRIT) figure

All acquired outcome measures are summarised in the Standard Protocol Items Recommendations for Interventional Trials (SPIRIT) figure (Fig. 1). The SPIRIT checklist is given in Additional file 1.

### Participant recruitment

Advertisements will be placed in local newspapers, noticeboards, and online social media outlets allowing participants to make initial contact directly with the research team via email. Furthermore, primary healthcare providers such as general practises in the greater Auckland region will be approached to engage with participant recruitment.

### Randomisation, blinding, and code-breaking

One member of our research team will perform computer-generated randomisation on the study day. Each patient’s weight will be obtained at the screening session allowing measurement of doses by the clinical team on each study day. Only the randomiser and medical doctor who delivers the intervention will know the identity of the drug to be delivered. None of these study team members will be involved in the psychiatric outcome assessments in order to maintain double-blind conditions. Further, the clinical team who perform psychiatric assessments will not be present during the interventions to avoid de-blinding. During patient debriefing, participants will be asked to identify which session they thought was the placebo and then informed as to the correct identification of sessions. However, in the event of acute deterioration of health requiring emergency hospital care (for example, in the case of severe anaphylaxis), the randomiser or medical doctor may provide un-blinding information regarding the identity of the delivered intervention.

### Alterations to allocated interventions

There will be no special criteria for discontinuing or modifying allocated interventions.

### Strategies to improve adherence

The study staff communicate with participants after the intervention day at 1 day, 3 days, 1 week, 2 weeks, 4 weeks, and 6 weeks. Study staff may use these telephone calls to remind participants along with email, phone call, or text to remind participants to adhere to study protocols (e.g., continue to wear Fitbits).

### Relevant concomitant care and post-trial care

Whilst participants are enrolled in the present clinical trial, there will not be any alteration to the access to usual care pathways for both trial arms. However, if a participant becomes ineligible for the present trial whilst enrolled, the participant may be discharged from the trial. Should a participant be discharged due to medical or psychiatric issues, the participant will be referred to relevant medical services. There is no anticipated harm for those who participate in the present trial. All participants will be reimbursed in supermarket vouchers for their time in the trial.

### Statistical analyses and power calculations

The principle methods of analysis for the present four parallel groups design will be one-way Analysis of Variance (ANOVA) and independent sample *t* tests. To avoid making assumptions about the normal distribution of the dependent variables, statistical resampling methods will be used to generate the null response distribution. Both intention to treat and per protocol analyses will be reported as per Consolidated Standards of Reporting Trials (CONSORT) guidelines [34].

The following power calculations for determining the antidepressant response of scopolamine were performed in G*Power 3.1 [35] with α = 0.05, 1 − β = 0.8. In order to establish clear a priori hypotheses around scopolamine’s antidepressant efficacy, an interim analysis will be conducted at *n* = 40 to compare the placebo group (*n*_*1*_ = 16) with all scopolamine groups (*n*_*2–4*_ = 24). This provides a minimum detectable effect size of *d*_*interim*_ = 0.92 (two-tailed, α = 0.05, 1 − β = 0.8, *n*_*1*_ = 16, *n*_*2–4*_ = 24). For an interim analysis where the conditional power is > 50%, the effect can be considered “promising” and sample size can be increased without biasing the final outcome analysis [36, 37]. At the interim analysis point (*n* = 40), conditional power of 50% would be achieved at *d* = 0.65, which would allow up to 80 participants to be recruited in theory. In practise, recruiting up to *n* = 20 more participants is feasible. At *n* = 60, the minimum effect size detectable is *d*_*final*_ = 0.75 (two-tailed, α = 0.05, 1 − β = 0.8, n_1_ = 24, n_2–4_ = 36). Hence, the stopping rule thresholds for effect sizes at *n* = 40 are:

1. *d*_*interim*_ > 0.92. Significant *p* < 0.05. Conclude that scopolamine hydrobromide has efficacious antidepressant properties.
2. *d*_*interim*_ < 0.75. Non-significant *p* value. Conclude that scopolamine hydrobromide does not have efficacious antidepressant properties.
3. 0.75 < *d*_*interim*_ < 0.92. Promising. Increase sample size by *n* = 20.
  - If *d*_*final*_ > 0.75 at *n* = 60, significant *p* < 0.05. Therefore, conclude that scopolamine hydrobromide has efficacious antidepressant properties.
  - If *d*_*final*_ < 0.75 at *n* = 60, non-significant *p* value. Therefore, conclude that scopolamine hydrobromide does not have efficacious antidepressant properties.

Prior studies of standard antidepressants in MDD patients have shown antidepressant effect sizes ranging from 0.5 to 1.1 in moderately and severely depressed patients, respectively [38]. Prior studies into scopolamine as an antidepressant demonstrated effect sizes of *d* = 3.4, 2.2, 1.7, and 1.2 [5, 6] in patients with MDD whereas the most recent study reporting no significant antidepressant effect exhibited an effect size of *d* = 0.58 [39]. The present study sample size (*n* = 60) is powered at *d* = 0.75 in order to detect ‘large’ effect size changes (defined as *d* > 0.8) [40]. These effect sizes represent depression rating scores which are effect size standardised and are in the region of past literature. The overall decision to recruit a maximum of *n* = 60 was limited by funding and practical constraints.

### Sub-group data analyses and handling missing data

Sub-group analyses, such as comparing the magnitude of the antidepressant response between genders, may be conducted depending on whether a significant antidepressant response is elicited. Depending on the extent and nature of missing data from a single participant, data may be excluded from final analyses or included using data imputation. For example, should a participant’s EEG data be unable to be collected after being randomised into the trial, the nature of the data makes it difficult to extrapolate; as such, the data from this participant would be excluded. However, should a participant miss the last MADRS structured interview (of the eight interviews conducted throughout the study), data imputation techniques such as last observation carried forward will be used.

### Adverse event reporting

All adverse events occurring during the trial will be recorded on the CRF and the GASE questionnaire, whether or not attributed to trial medication. All suspected unexpected serious adverse reactions and severe adverse events will be reported to the New Zealand Medicines and Medical Devices Safety Authority ‘MedSafe’.

### Data and Safety Monitoring Committee

The Data and Safety Monitoring Committee for these trials will comprise two independent medical practitioners with specialisations in anaesthesiology and psychiatry as well as a biostatistician.

**Table:** 

Dr. Gemma Malpas Consultant Anaesthetist Auckland City Hospital	Dr Adib Essali Consultant Psychiatrist Counties Manukau District Health Board	Dr Alana Cavadino Biostatistician Faculty of Medical and Health Sciences, University of Auckland

In the unlikely event of a severe adverse event being reported, the Data and Safety Monitoring Committee may decide to suspend the trial or request suspension until the research protocol is appropriately revised. From commencement of the trial until its termination the Data and Safety Monitoring Committee will be provided recruitment, outcome, and adverse effect data on a 6-monthly basis. Stopping decisions will be made in consultation with the Data and Safety Monitoring Committee.

### Data management and record keeping

Separate paper-based case report files (CRFs) will be kept for each participant and completed in a neat, clearly legible manner. Demographics, medical history, height, weight, and current medications will be entered directly into the CRF. All electronic data will be stored on secure University of Auckland servers which include password protection, multi-site backups and tape archiving. An original, unprocessed version of every data file will be kept on the servers such that these files may only be modified by a University of Auckland Information Technologies systems administrator, thus ensuring the fidelity and audit capability of all electronic data. Scanned versions of all paper-based CRFs and source data formats will be made and held on the servers in password protected files to ensure fidelity of these data and allowing future audit of extracted data.

Participants will be identified by a unique trial-specific number and/or code in any electronic database. The name and any other identifying detail will not be included in any trial data electronic file. On all trial-specific documents, other than the signed consent and page one of the CRF (separately filed), the participant will be referred to by the trial participant code, not by name. Paper-based data will be held for a period of 10 years from the completion of the trial.

### Dissemination policy

The results from the present trial will be published in specialised academic journals. Furthermore, results in an easy to read format may be distributed through news media or social media outlets. Participants are able to request a summary of their personal results.

## Discussion

The present study will characterise the antidepressant and physiological effects of a single intravenous dose infusion of scopolamine hydrobromide. The study aims to evaluate the antidepressant efficacy of scopolamine, the optimal scopolamine dose, and the duration of scopolamine’s antidepressant effect and to test the efficacy of glycopyrrolate in maintaining superior blinding to saline. The overall purpose is to determine the parameters for designing future crossover trials to better understand the underlying antidepressant mechanisms of scopolamine. Determining the washout period of the antidepressant effects of scopolamine can be used to inform ideal temporal spacing of administered active and placebo drugs for crossover studies along with potentially informing clinical best practice for future repeat administrations to sustain the antidepressant response in the long term. Additionally, the use of an active placebo is an important experimental consideration to reduce unblinding effects, thereby allowing for a statistically cleaner determination of antidepressant effect size.

By collecting EEG in the acute phases of scopolamine administration, the alpha modulating effects of scopolamine can be measured. Given that alpha power has been shown to be elevated in MDD patients [41–43] and that excess alpha power is associated with a favourable response to antidepressant therapies [44], it is particularly interesting to see whether alpha modulating effects of scopolamine may facilitate the antidepressant response.

One of the major issues we anticipate in the design of this trial is failure to replicate the antidepressant properties of scopolamine. In order to properly establish this, we have designed clear stopping rules. We do not anticipate other practical or operational issues in conducting this study.

### Trial status

The study started advertising for enrolment on 24 July 2019. The first participant was enrolled on 12 August 2019. Enrolment is expected to be completed in August 2020. The protocol version is 1.5, August 2019.

## Supplementary information

**Additional file 1.** SPIRIT 2013 checklist: Recommended items to address in a clinical trial protocol and related documents.

## Data Availability

De-identified datasets generated from the present study will be made available from the corresponding author on reasonable request. The model consent form and other related documentation are available from the corresponding author upon reasonable request. However, the physical collection of biological specimens (i.e., blood samples) is limited to the purposes outlined in the “Study design” section and will not be made available for other purposes or ancillary studies.

## Abbreviations

5D-ASC: 5-Dimensional Altered State of Consciousness Rating Scale
ANOVA: Analysis of Variance
BAES: Biphasic Alcohol Effects Scale
CADSS: Clinician-Administered Dissociative States Scale
CEQ: Credibility Expectancy Questionnaire
CONSORT: Consolidated Standards of Reporting Trials
DSM-V: *Diagnostic and Statistical Manual* edition 5
ECG: Electrocardiography
EDTA: Ethylenediaminetetraacetic acid
EEG: Electroencephalography
GASE: General Assessment of Side Effects
MADRS: Montgomery–Åsberg Depression Rating Scale
MDD: Major depressive disorder
MINI: Mini-International Neuropsychiatric Interview
QIDS: uick Inventory of Depression Scale
SHAS: Subjective High Assessment Scale
SPIRIT: Standard Protocol Items Recommendations for Interventional Trials
SSRI: Selective serotonin reuptake inhibitor
VAS: Visual acuity scale

## References

[1] World Health Organisation (2018). Depression.

[2] American Psychiatric Association. Diagnostic and Statistical Manual of Mental Disorders (DSM-5®). 5th ed. Arlington: American Psychiatric Association; 2013.

[3] Warden D, Rush AJ, Trivedi MH, Fava M, Wisniewski SR (2007). The STAR*D Project results: a comprehensive review of findings. Curr Psychiatry Rep.

[4] Gupta S, Gersing KR, Erkanli A, Burt T (2016). Antidepressant regulatory warnings, prescription patterns, suicidality and other aggressive behaviors in major depressive disorder and anxiety disorders. Psychiatr Q.

[5] Furey ML, Drevets WC (2006). Antidepressant efficacy of the antimuscarinic drug scopolamine: a randomized placebo-controlled clinical trial. Arch Gen Psychiatry.

[6] Drevets WC, Furey ML (2010). Replication of scopolamine’s antidepressant efficacy in major depressive disorder: a randomized, placebo-controlled clinical trial. Biol Psychiatry.

[7] Furey ML, Khanna A, Hoffman EM, Drevets WC (2010). Scopolamine produces larger antidepressant and antianxiety effects in women than in men. Neuropsychopharmacology.

[8] Ellis JS, Zarate CA, Luckenbaugh D, Furey ML (2014). Antidepressant treatment history as a predictor of response to scopolamine: clinical implications. J Affect Disord.

[9] Park L, Furey M, Nugent AC, Farmer C, Ellis J, Szczepanik J (2018). Neurophysiological changes associated with antidepressant response to ketamine not observed in a negative trial of scopolamine in major depressive disorder. Int J Neuropsychopharmacol.

[10] Xu Ying, Hackett Maree, Carter Gregory, Loo Colleen, Gálvez Verònica, Glozier Nick, Glue Paul, Lapidus Kyle, McGirr Alexander, Somogyi Andrew A., Mitchell Philip B., Rodgers Anthony (2015). Effects of Low-Dose and Very Low-Dose Ketamine among Patients with Major Depression: a Systematic Review and Meta-Analysis. International Journal of Neuropsychopharmacology.

[11] Liem-Moolenaar M, de Boer P, Timmers M, Schoemaker RC, van Hasselt JG, Schmidt S (2011). Pharmacokinetic-pharmacodynamic relationships of central nervous system effects of scopolamine in healthy subjects. Br J Clin Pharmacol.

[12] Walsh BT, Seidman SN, Sysko R, Gould M (2002). Placebo response in studies of major depression: variable, substantial, and growing. JAMA.

[13] Ebert U, Grossmann M, Oertel R, Gramatte T, Kirch W (2001). Pharmacokinetic-pharmacodynamic modeling of the electroencephalogram effects of scopolamine in healthy volunteers. J Clin Pharmacol.

[14] Ebert U, Kirch W (1998). Scopolamine model of dementia: electroencephalogram findings and cognitive performance. Eur J Clin Investig.

[15] Bruder GE, Stewart JW, Tenke CE, McGrath PJ, Leite P, Bhattacharya N (2001). Electroencephalographic and perceptual asymmetry differences between responders and nonresponders to an SSRI antidepressant. Biol Psychiatry.

[16] Bruder GE, Sedoruk JP, Stewart JW, McGrath PJ, Quitkin FM, Tenke CE (2008). EEG alpha measures predict therapeutic response to an SSRI antidepressant: pre and post treatment findings. Biol Psychiatry.

[17] Blain-Moraes S, Lee U, Ku S, Noh G, Mashour GA (2014). Electroencephalographic effects of ketamine on power, cross-frequency coupling, and connectivity in the alpha bandwidth. Front Syst Neurosci.

[18] Kochs E, Scharein E, Mollenberg O, Bromm B, am Esch JS (1996). Analgesic efficacy of low-dose ketamine. Somatosensory-evoked responses in relation to subjective pain ratings. Anesthesiology.

[19] Rivolta D, Heidegger T, Scheller B, Sauer A, Schaum M, Birkner K (2015). Ketamine dysregulates the amplitude and connectivity of high-frequency oscillations in cortical-subcortical networks in humans: Evidence from resting-state magnetoencephalography-recordings. Schizophr Bull.

[20] Muthukumaraswamy SD, Shaw AD, Jackson LE, Hall J, Moran R, Saxena N (2015). Evidence that subanesthetic doses of ketamine cause sustained disruptions of NMDA and AMPA-mediated frontoparietal connectivity in humans. J Neurosci.

[21] Thase ME, Rush AJ (1997). When at first you don’t succeed: sequential strategies for antidepressant nonresponders. J Clin Psychiatry.

[22] Williams JB, Kobak KA (2008). Development and reliability of a structured interview guide for the Montgomery Asberg Depression Rating Scale (SIGMA). Br J Psychiatry.

[23] Rush AJ, Trivedi MH, Ibrahim HM, Carmody TJ, Arnow B, Klein DN (2003). The 16-Item Quick Inventory of Depressive Symptomatology (QIDS), clinician rating (QIDS-C), and self-report (QIDS-SR): a psychometric evaluation in patients with chronic major depression. Biol Psychiatry.

[24] Bowdle TA, Radant AD, Cowley DS, Kharasch ED, Strassman RJ, Roy-Byrne PP (1998). Psychedelic effects of ketamine in healthy volunteers. Anesthesiology.

[25] Rief W, Glombiewski JA, Barsky AJ (2009). Generic assessment of side effects.

[26] Koželj G, Perharič L, Stanovnik L, Prosen H (2014). Simple validated LC–MS/MS method for the determination of atropine and scopolamine in plasma for clinical and forensic toxicological purposes. J Pharm Biomed Anal.

[27] Judd LL, Hubbard RB, Huey LY, Attewell PA, Janowsky DS, Takahashi KI (1977). Lithium carbonate and ethanol induced highs in normal subjects. Arch Gen Psychiatry.

[28] Martin CS, Earleywine M, Musty RE, Perrine MW, Swift RM (1993). Development and validation of the biphasic alcohol effects scale. Alcohol Clin Exp Res.

[29] Bremner JD, Krystal JH, Putnam FW, Southwick SM, Marmar C, Charney DS (1998). Measurement of dissociative states with the Clinician-Administered Dissociative States Scale (CADSS). J Trauma Stress.

[30] Dittrich A (1998). The standardized psychometric assessment of altered states of consciousness (ASCs) in humans. Pharmacopsychiatry.

[31] Rutherford BR, Marcus SM, Wang P, Sneed JR, Pelton G, Devanand D (2013). A randomized, prospective pilot study of patient expectancy and antidepressant outcome. Psychol Med.

[32] Gillin JC, Sutton L, Ruiz C, Darko D, Golshan S, Risch SC (1991). The effects of scopolamine on sleep and mood in depressed patients with a history of alcoholism and a normal comparison group. Biol Psychiatry.

[33] Adrien J (2002). Neurobiological bases for the relation between sleep and depression. Sleep Med Rev.

[34] Schulz KF, Altman DG, Moher D (2010). CONSORT 2010 Statement: updated guidelines for reporting parallel group randomised trials. BMJ.

[35] Faul F, Erdfelder E, Lang AG, Buchner A (2007). G*Power 3: a flexible statistical power analysis program for the social, behavioral, and biomedical sciences. Behav Res Methods.

[36] Chen YHJ, DeMets DL, Lan KKG (2004). Increasing the sample size when the unblinded interim result is promising. Stat Med.

[37] Chen YJ, Li C, Lan KG (2015). Sample size adjustment based on promising interim results and its application in confirmatory clinical trials. Clin Trials.

[38] Khan A, Brodhead AE, Kolts RL, Brown WA (2005). Severity of depressive symptoms and response to antidepressants and placebo in antidepressant trials. J Psychiatr Res.

[39] Park L, Furey M, Nugent AC, Farmer C, Ellis J, Szczepanik J (2019). Neurophysiological changes associated with antidepressant response to ketamine not observed in a negative trial of scopolamine in major depressive disorder. Int J Neuropsychopharmacol.

[40] Sullivan GM, Feinn R (2012). Using effect size—or why the p value is not enough. J Grad Med Educ.

[41] Begić D, Popović-Knapić V, Grubišin J, Kosanović-Rajačić B, Filipčić I, Telarović I, Jakovljević M (2011). Quantitative electroencephalography in schizophrenia and depression. Psychiatr Danub.

[42] Jaworska N, Blier P, Fusee W, Knott V (2012). Alpha power, alpha asymmetry and anterior cingulate cortex activity in depressed males and females. J Psychiatr Res.

[43] Grin-Yatsenko VA, Baas I, Ponomarev VA, Kropotov JD (2010). Independent component approach to the analysis of EEG recordings at early stages of depressive disorders. Clin Neurophysiol.

[44] Ulrich G, Renfordt E, Zeller G, Frick K (1984). Interrelation between changes in the EEG and psychopathology under pharmacotherapy for endogenous depression. Pharmacopsychiatry.

